# Protocol for a scoping review study on the prevalence and public health consequences of non-medical use (NMU) of tramadol in Africa

**DOI:** 10.1371/journal.pone.0285809

**Published:** 2023-05-19

**Authors:** Saidou Sabi Boun, Olumuyiwa Omonaiye, Sanni Yaya

**Affiliations:** 1 School of International Development and Global Studies, University of Ottawa, Ottawa, Ontario, Canada; 2 Centre for Quality and Patient Safety Research, Institute for Health Transformation, Deakin University, Geelong, Australia; 3 Deakin University Centre for Quality and Patient Safety Research – Eastern Health Partnership, Box Hill, Victoria, Australia; 4 The George Institute for Global Health, Imperial College London, London, United Kingdom; Xiamen University - Malaysia Campus: Xiamen University - Malaysia, MALAYSIA

## Abstract

**Background:**

Tramadol is one of the most prescribed painkillers in the world. It is a synthetic opioid that is an excellent alternative to morphine and its derivatives in African countries. It is an essential drug due to its low cost and constant availability. However, the health consequences of tramadol use due to illicit trafficking, like those caused by fentanyl and methadone in North America, are poorly documented. This scoping review aims to understand the nature and extent of the use and health consequences of the Non-Medical Use (NMU) of tramadol in Africa to guide future research.

**Methods:**

Due to the perceived lack of African literature on the subject, our search strategy is based on the simultaneous use of the keywords "tramadol" and Medical Subject Heading (MeSH), such as "Drug abuse," "illicit drugs," or "Prescription Drug Misuse," combined with the term "Africa" and Boolean operators (and, or not) to form our search equations. Two researchers will independently select studies from literature searched in several databases such as Medline, Embase, the Scopus database, Web of Science, the African Journals online database, and for grey literature Google Scholar without any time restriction. All research, in various formats, conducted in Africa, will be included in our study on the prevalence of use in different African population groups or on evidence of addiction, intoxication, seizures and mortality related to NMU of tramadol.

**Results:**

Through this study, we aim to map consumers and identify risk factors, health consequences, and prevalence of the NMU of tramadol in African countries.

**Discussion:**

We are conducting the first scoping review study to investigate the prevalence and consequences of NMU of tramadol in Africa. Upon completion, our findings will be published in a peer-reviewed journal and presented at relevant conferences and workshops. However, since health is not limited to the lack of disease, our study is likely incomplete without incorporating the studies of the social impact of NMU of tramadol.

**Systematic review registration:**

Open Science Framework: https://osf.io/ykt25/.

## Background

Tramadol is one of the most prescribed painkillers in the world [1]. It is a synthetic opioid used for moderate to severe pain and is an excellent alternative to morphine due to its consistent availability and low price [2].

Initially, it was assessed as having common addictive properties, so it has not been placed under international control, like fentanyl and methadone, which are responsible for the opioid crisis in other parts of the world. In addition, physicians prefer it to non-steroidal anti-inflammatory drugs, as it is more tolerable and has fewer side effects [2, 3]. Tramadol is widely used for neuropathic and nociceptive pain, back pain, and people with cancer and is often prescribed for the pain of sickle cell anemia [4, 5]. It is considered an essential medicine and is included in the essential and generic medicines (EGM) list of many African countries, such as Botswana, Niger, Ghana, Mali, Côte d’Ivoire, Ghana, and Nigeria [2].

Several articles have reported an increase in the non-medical use (NMU) of tramadol, with the presence of addiction symptoms similar to those of morphine, especially when taken regularly at doses above therapeutic levels; however, this is becoming increasingly common [6–12]. Thus, in a few years, tramadol has become a public health problem in Africa in the same way as fentanyl has been responsible for the opioid crisis in the United States [13, 14]. Moreover, the enormous amounts of illicit drugs seized by the authorities in African countries establish a growing interest in this product, especially among the younger populations. For example, in 2014, 17 tonnes of smuggling were seized across West African countries, 121 tonnes in 2015 and 170 tonnes in 2017 [2]. The NMU of tramadol is all the more harmful as the illicit doses sold on the pavements and markets are 2 to 5 times higher than the usual doses (100 to 250 mg against 50 mg usually), thus increasing its addictive power [10, 11]. There are several reasons for the sudden increase in the popularity of tramadol among African youth. For example, the tablet is available everywhere in West Africa, in every "pharmacy on the floor.”

Unlike other products, it is sold in full view of health and political authorities. In addition, tramadol is sold for between 0.20 and 1 USD per 10-tablet pack, which is far less than other drugs [2]. This explains why it is described as the ’cocaine of the poor’ by some authors [11]. Conversely, compared to other drugs, marijuana is easier to transport and conceal, and the punishments incurred by sellers and possessors are less severe, leading to the population perceiving it as a medicine [15]. In most African countries, it is easy to obtain in pharmacies without a medical prescription [2]. In North African countries, a literature review showed that tramadol is the second most commonly used drug by students in Egypt due to its psychoactive properties [16] and more generally in countries in the Eastern Mediterranean region, including Libya, Morocco, Somalia and Tunisia [17]. In Central Africa, especially Cameroon, tramadol is used illicitly for its psychoactive properties and to increase work efficiency [18].

Tramadol is usually taken with tea, coffee, or alcohol, with other pharmaceutical drugs, such as benzodiazepines, in several African countries [9, 10]. It is consumed collectively during weddings, baptisms, during community work, for euphoric sensation seeking, which is one of the main reasons for its consumption [15]. It is also taken individually to combat fatigue by manual workers or in the search for physical or sexual performance [14, 15]. Its use is often associated with public disorder and traffic accidents due to potential side effects like dizziness, euphoria, and changes in fear and pain [15]. Due to the increased NMU of tramadol in many African countries, some authors describe the situation as an opioid crisis due to tramadol [13].

Despite the warning signs and numerous press articles on the health and social consequences of the NMU of tramadol [19–22], more scientific studies are needed to circumscribe the extent and understand the health consequences of the phenomenon, as has been done in many parts of the world [23, 24]. Therefore, our scoping study aims to understand the nature and extent of tramadol NMU use and its health consequences in Africa to guide future research.

## Methods

### Protocol design

**Fig 1:**
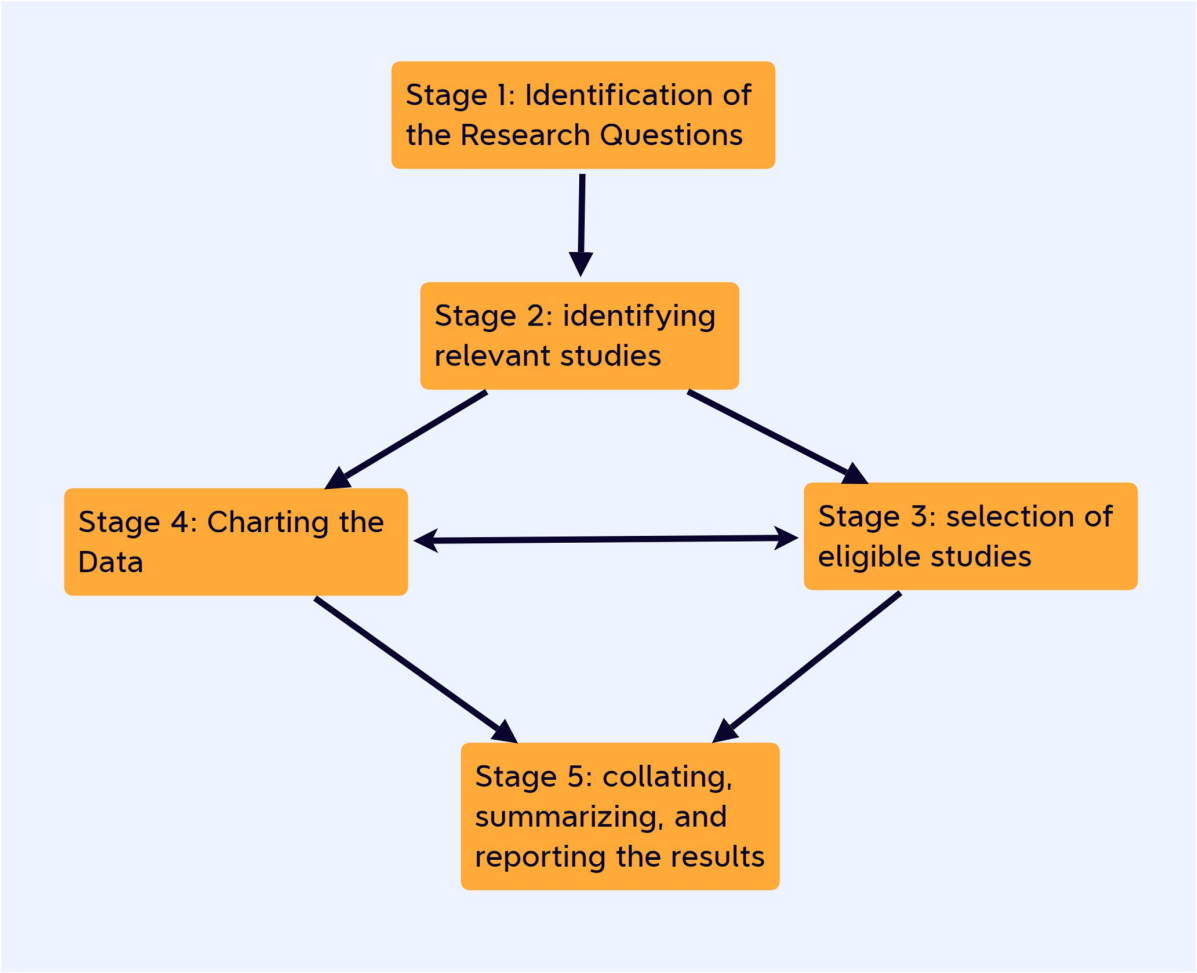
Scoping review research methodology inspired by Arksey and O’Malley (2005).

The study will be conducted according to the five methodological steps defined by Arksey and O’Malley (2005) and modified by Joanna Briggs Institute guidelines for conducting a scoping review to ensure systematic and repeatable studies [25]. The five steps are: 1) formulate the research question; 2) identify relevant studies; 3) select studies according to inclusion and exclusion criteria; 4) extract and map the results; 5) report the results [26] (Fig 1). The PRIMA-SCR (Preferred Reporting Items for Systematic Reviews and Meta-Analysis Extension for Scoping Reviews) checklist will be used to report, filter and communicate our results [27].

### Stage 1: Identification of the research questions

The overall objective of this study is to explore the body of knowledge related to the prevalence and health consequences of NMU of tramadol in African countries. Consequently, we will conduct an exploratory synthesis of published work related to the prevalence, type of population involved and health consequences of NMU of tramadol. Considering that the research question is the starting point for our scoping study, we have formulated two research questions in order to achieve our objectives:

1. What is the prevalence of NMU of tramadol in the most at-risk subpopulations in Africa?
2. What are the related risk factors and health consequences associated with the NMU of tramadol in Africa?

### Stage 2: Identifying relevant studies

This scoping review will be conducted using the PCC (Population, Concept, Context) framework recommended by JBI [28]. Our search strategy will be based on the PCC framework described in Table 1.

**Table 1:** PCC framework.

PCC element	Definition	Example
Population	African population (no restriction)	Nigerian, Beninese, South African, Burkina Faso people, Malian, Egyptian, etc.
Concept	Tramadol non-medical use	Extracting prevalence of NMU of tramadol among African subcategories population, risk factors and public health consequences due to tramadol NMU
Context	Health research	Public Health

The literature search will be done in several databases such as Medline, Embase, Scopus databases, the Web of Science, the African Journal online database, and for grey literature Google Scholar. Given the supposed weakness of the African literature on the subject, the keyword "tramadol," without any time restriction, will be used to query the selected databases. To this word will be added Medical Subject Heading (MeSH) as "Drug abuse," "illicit drugs," or "Prescription Drug Misuse," and "Africa." These keywords combined with the Boolean operators (and, or, not) will result in search equations that will form the basis of our search strategy.

### Stage 3: Selection of eligible studies

All articles in the scientific and grey literature related to the research question, our research framework, meeting our inclusion criteria and available in English or French in the selected databases will be included in the study. Our study will include all research conducted in Africa on the prevalence of NMU of tramadol in various population groups, evidence of addiction, intoxication, seizures, and mortality related to NMU of tramadol in a variety of formats such as original research, reviews, case reports, cohort or retrospective studies, and credible reports from international organizations involved in the control of NMU of tramadol.

All the articles derived from our search process will be uploaded to bibliographic data management software, such as EndNote 20, to delete duplicate research. The online Covidence platform will facilitate the screening of references [29], and two researchers will independently screen references at the title, abstract, and full-text screening stages. Any disagreements about the inclusion or exclusion of a study will be resolved by consensus, and when no agreement is reached, the third researcher will be consulted. The review will exclude: 1) studies without a specific indicator (prevalence, mortality, morbidity, etc.); 2) interventional and quasi-experimental studies because these studies involved tramadol NMU exposure manipulation. Also, our scoping review is not intended to include studies done in vitro to test tramadol addiction or toxicity; 3) studies in which tramadol is reported in combination with other drugs in such a way that it is not possible to specify isolated effects of tramadol; and 4) Texts and opinion literature.

### Stage 4: Charting the data

Two researchers will independently use the Covidence platform for data extraction and mapping; if there is any disagreement, a third researcher will be consulted. A matrix of data extraction will be created to focus on data that will help answer our research questions and could include, for example:

1. The author’s name and year of publication.
2. Country of study.
3. Target population.
4. Type of study.
5. Study design.
6. Risk factors identified.
7. Main results about the prevalence or health consequences of tramadol NMU.

Our study’s results can be utilized for future investigations into the NMU of tramadol among the population groups most impacted by the phenomenon.

### Stage 5: Collating, summarizing, and reporting the results

As this is a scoping review, the quality of the included studies will not be assessed by the practice advocated by JBI and several other authors [25, 28, 30]. Our scoping review aims to explore the fields of knowledge related to the prevalence and the African subpopulations concerned by the phenomenon and to map the overall health consequences of tramadol NMU.

Prevalence will be reported narratively and linked to the categories of subpopulations concerned by tramadol NMU. The qualitative thematic analysis will allow us to answer the questions relative to the health consequence of tramadol NMU. Using the WHO International Classification of Diseases-11 (ICD-11), we identified three categories of medical conditions related to tramadol use:

1. Intoxication is the set of conditions that follows the ingestion of tramadol, resulting in disturbances in the level of consciousness, cognition, perception, behaviour or other psycho-physiological function and responses. The troubles are directly linked to the pharmacological effect of tramadol and resolve with time, except where the medical complication occurs.
2. Dependence syndrome: a cluster of behavioural, cognitive, and physiological phenomena that develop after repeated substance use. This includes a strong desire to take tramadol, difficulties controlling its use, persisting in its benefits despite harmful consequences, and prioritizing tramadol over other activities and obligations.
3. Withdrawal symptoms: a group of variable clustering and severe symptoms occurring on absolute or relative tramadol withdrawal after persistent NMU. The onset and course are related to tramadol and the dose used immediately before cessation or reduction of use. Medical complications may complicate the withdrawal symptoms.

## Results

**Fig 2:**
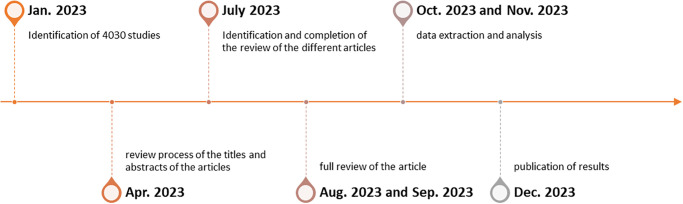
Timeline of expected results.

In January 2023, the search strategy used for this study identified 4030 studies in the selected databases. Two independent researchers will conduct the review process of the titles and abstracts of the articles on the COVIDENCE platform in the last week of April 2023. The identification and completion of the review of the different articles are expected to occur by the end of July 2023. Subsequently, the full review of the articles that have passed the initial selection will commence in August and September 2023, followed by the data extraction and analysis stage in October and November 2023. The study results are anticipated to be available and prepared for publication by December 2023 (Fig 2).

## Discussion

The scoping review aims to investigate the prevalence, subpopulations affected, and health consequences of non-medical use of tramadol in African countries. To do this, two types of data will be gathered: quantitative data to show the phenomenon’s prevalence in different subpopulations and qualitative data to illustrate the health consequences of the NMU of tramadol. This study will not require approval from an ethics committee as the Scoping Review methodology involves reviewing and collecting data from publicly available materials. To disseminate the results, a thematic analysis will be conducted to better understand tramadol NMU’s health consequences. The results will be submitted for publication in a scientific journal and presented at relevant conferences such as the International Conference on Public Health in Africa (CPHA).

We anticipate certain limitations in our review. Firstly, it is possible that many locally conducted studies, which have not been published yet, may not be incorporated in this review. Secondly, since the phenomenon under study is hidden, the data collected through self-reporting or prevalence surveys may be prone to underreporting or social desirability bias. Furthermore, health is not only the absence of disease; thus, the consequences of the NMU of tramadol extend beyond health implications. The United Nations Office against Drugs and Crime (UNODC) has also highlighted tramadol NMU’s role in undermining the Sahel’s economies and security [14]. This link has been further confirmed by the Institute for Security Studies report, which focuses on the relationship between violent extremism and illicit activities in the so-called 3-border zone between Burkina Faso, Niger and Mali [31]. According to this report, two cases emerge from the attitude of armed groups regarding the nature or degree of their involvement in illicit activities: 1) they are sometimes beneficiaries of trafficking products such as tramadol, used as a stimulant for fighters of armed groups; 2) without being involved, they may derive income from illicit trafficking activities by levying taxes on convoys in their area of control, providing armed escorts, protection or transport of tramadol traffickers. Future research into the consequences of NMU of tramadol should include the social and economic impacts on the African population.

## Study implications

By exploring the prevalence and public health consequences of the NMU of tramadol, this study can contribute to a better understanding of the scale of the issue and inform the development of evidence-based interventions and policies to address it. Additionally, the findings of this study can serve as a valuable resource for future research on the NMU of tramadol in Africa, providing insights into knowledge gaps and areas that require further investigation. Ultimately, the results of this study have the potential to positively impact public health outcomes and contribute to the overall well-being of individuals and communities affected by the NMU of tramadol in Africa.

## Limitations

The present study may encounter potential limitations, including (1) language bias as only studies published in English and French will be included, which may constrain the scoping review and disregard relevant studies published in other languages; (2) publication bias, as only studies that have been published will be considered, thereby missing unpublished studies or studies in progress; (3) quality of studies, since the scoping review will not evaluate the quality of the included studies, the results may be impacted by the quality of the studies and the likelihood of bias in their design and methodology; (4) heterogeneity of studies, since the studies included in the review may exhibit variations in their definitions of NMU of tramadol and their data collection methods, making it challenging to make direct comparisons across studies. Lastly, it is noteworthy that this study does not include articles addressing the economic and social ramifications of NMU of tramadol, which may extend beyond health-related consequences.

## Conclusion

This scoping review aims to identify and map the existing literature on the prevalence and public health consequences of the NMU of tramadol in Africa. It will focus on the prevalence, patterns, and risk factors of tramadol misuse and the public health consequences of its NMU. In this protocol, we identified a comprehensive search strategy and data extraction process to gather relevant studies for the review. However, we also highlighted potential limitations, including language bias, publication bias, heterogeneity of studies, and limited assessment of study quality. Despite these limitations, this scoping review has the potential to provide valuable insights into the NMU of tramadol in Africa and its public health implications.

## Supporting information

S1 File Pilot search strategy. (DOCX)
