# Supplementary material for: Protocol for a scoping review study on the prevalence and public health consequences of non-medical use (NMU) of tramadol in Africa
Source: PLoS One. 2023 May 19;18(5):e0285809. doi: 10.1371/journal.pone.0285809 (PMC10198492; doi:10.1371/journal.pone.0285809)
Supplement: S1 File — (DOCX) [file pone.0285809.s001.docx]

|  | Search string | Data of Search | Number of results |
| --- | --- | --- | --- |
| PubMed/Medline | (tramadol[Title/Abstract]) AND (Africa) | 19-janv-23 | 103 |
| OVID Plateform (Embase) Classic+Embase | (tramadol[Title/Abstract]) AND (Africa) | 20-janv-23 | 2 |
| Web of Science | ("tramadol") (Topic) AND africa (All Fields) | 06-janv-23 | 72 |
| Scopus | ( TITLE ( tramadol ) AND TITLE-ABS-KEY ( ( "abuse" OR "non-medical use" OR "abuse" ) ) AND ALL ( africa ) ) | 06-janv-23 | 15 |
| African Journal Online Database | ("tramadol") AND ("Non-medical use" OR "misuse" OR "abuse*") | 19-janv-23 | 178 |
| Google Scholar | ("tramadol") AND ("Non-medical use" OR "misuse" OR "abuse*") AND ("Africa") | 20-janv-23 | 3660 |
